# Minimally invasive closure of a progressive pseudoaneurysm of the ascending aorta: A case report

**DOI:** 10.3389/fcvm.2023.1134196

**Published:** 2023-03-20

**Authors:** Yuan Wu, Linglin Fan, Fei Liu, Hui Zhuang, Xijie Wu

**Affiliations:** ^1^ Department of Cardiac Surgery, Xiamen Cardiovascular Hospital of Xiamen University, School of Medicine, Xiamen University, Xiamen, China; ^2^ Department of Vascular Surgery, Xiamen Cardiovascular Hospital of Xiamen University, School of Medicine, Xiamen University, Xiamen, China

**Keywords:** aorta, pseudoaneurysm, minimally invasive treatment, high-risk patients, mortality

## Abstract

Ascending aortic pseudoaneurysm (AAP) is rare but may cause life-threatening complications. Although the placement of a stent graft and the use of occluder devices and vascular plugs to exclude pseudoaneurysm are adopted for some patients, the management of progressive pseudoaneurysms that may rupture at any time remains a challenge that needs to be addressed. In this study, we present the case of a patient with an AAP that was caused by aortic and mitral valve replacement for the giant left ventricle. Aortic pseudoaneurysm was suspected on the basis of a spherical cystic echo (70 × 80 mm) of the ascending aorta; this pseudoaneurysm was detected by an ultrasonic cardiogram, and the diagnosis was confirmed by an aortic computed tomography angiography (CTA) examination. To prevent the unexpected rupture of a progressive pseudoaneurysm, our patient was treated with a 28- mm ASD occluder without any procedural complications. Our patient has a good prognosis, which will inspire clinicians to choose minimally invasive procedures when dealing with such high-risk cases in emergency situations.

## Background

Ascending aortic pseudoaneurysm (AAP) is a rare condition that occurs in less than 0.5% of all cardiothoracic surgical patients (1). If a large pseudoaneurysm is located in the retrosternal space, then there is a very high risk of massive bleeding from rupture during resternotomy (2). Redo surgery requires lengthy and meticulous tissue dissection with significantly high mortality and morbidity (3). We performed a minimally invasive closure on a patient with ascending aortic pseudoaneurysm to prevent the development of this complication.

## Case presentation

**Figure 1:**
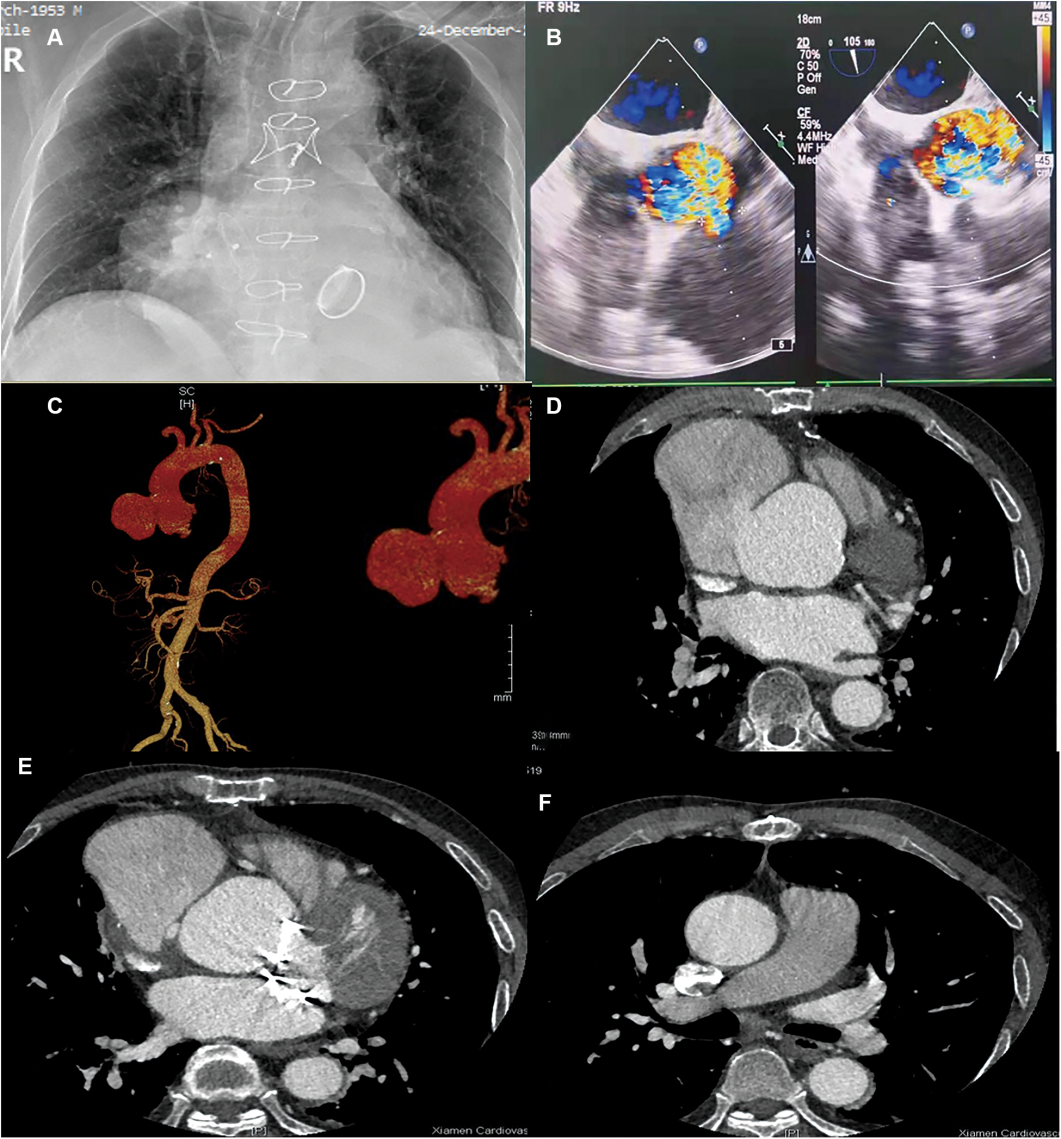
Preoperative: (**A**) Chest ray; (**B**) Cardiac ultrasound; (**C–F**) CT of coronary artery + thoracic and abdominal aorta [(**C**) Front position; (**D**) Tumor waist; (**E**) Lower border of the tumor; (**F**) Pulmonary bifurcation].

A 68-year-old male was asymptomatic until a postoperative review of calf lacerations was performed. A 4/6 grade of diastolic whistling murmur in the auscultation area around the mitral valve was detected on physical examination. His history was significant for aortic valve and mitral valve replacement surgery in 2017 due to severe aortic and mitral regurgitation. A chest x-ray indicated an enlarged, tumor-like swelling in the right cardiac border and a metal shadow (Figure 1A). Routine blood tests revealed moderate anemia with a hemoglobin level of 77 g/L, a CRP level of 6.29 mg/L, and an albumin level of 32.50 g/L. During hospitalization, the D-dimer level was approximately 6.96 mg/L and there was persistently elevated calcitoninogen, which indicated that a pseudoaneurysm may rupture at any time leading to sudden death. In addition, he suffered serious damage to his liver and kidney function. Other laboratory test results were normal. A cardiac ultrasound indicated that the breadth of the aortic sinus was approximately 63 mm, the sinus junction had disappeared, the aortic arch diameter was 37 mm, and the descending aortic diameter was 28 mm (Figure 1B). A CT of the coronary arteries + thoracic and abdominal aorta suggested that the ascending aorta and aortic sinus were widened, the latter to an area of 49 mm × 50 mm. The right anterior border of the ascending aorta presented with an aortic aneurysm–like structure measuring approximately 72 mm × 84 mm (Figures 1C–F). From the image, we can see that the breaking mouth from the left crown is 5.3 cm, and the breaking mouth from the right crown is 5.2 cm, with a tumor neck of 4.01 cm (Supplementary Material S1).

**Figure 2:**
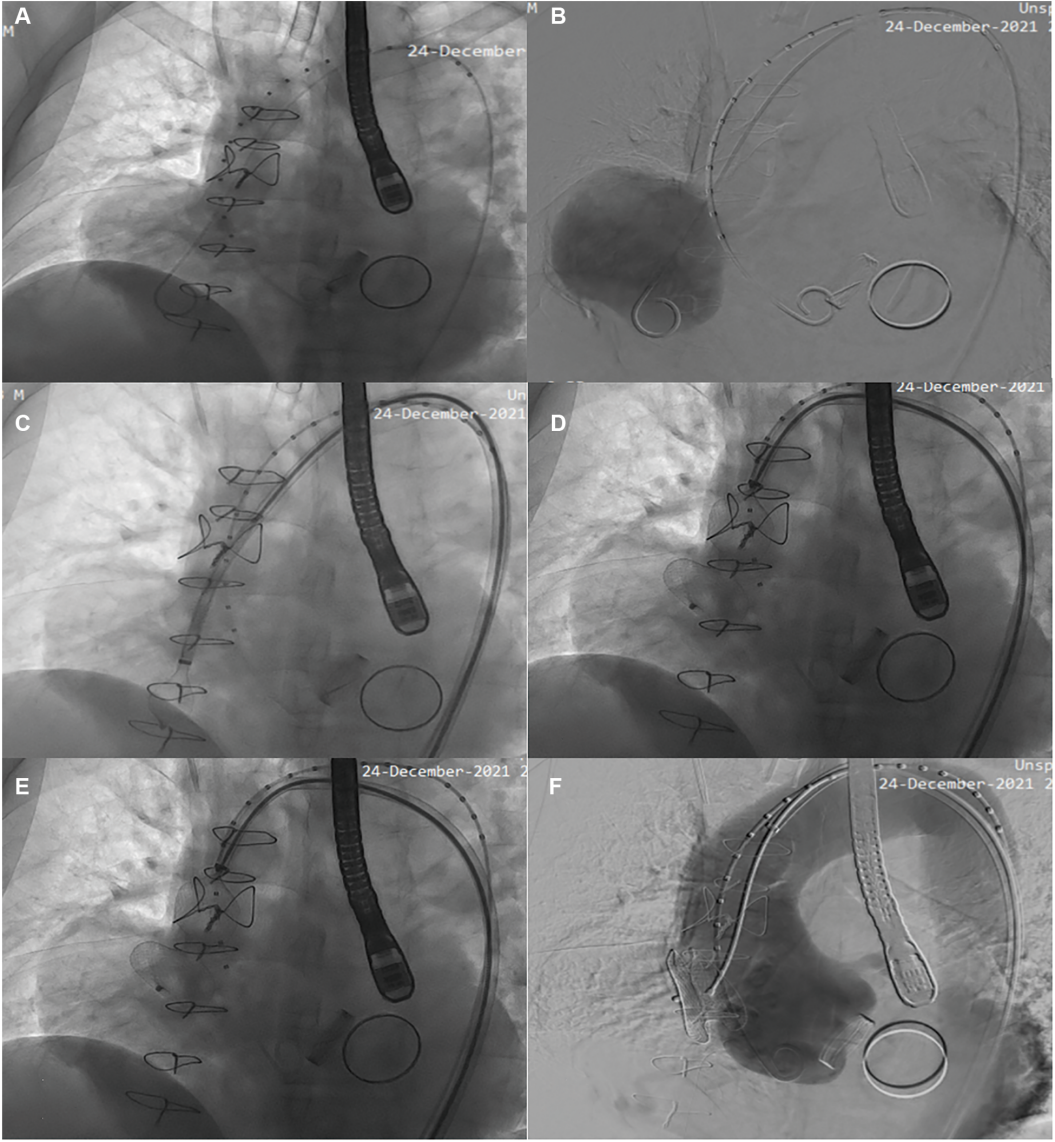
Angiography suggested that the shape of the ascending aortic sinus and the left and right coronary arteries were normal, that there was a crack at the junction of the ascending aorta, and of tumor-like deposits of a contrast agent (**A,B**). First, the blood pressure was reduced to approximately 70/40 mmHg. Then, a Lunderquist guidewire was fixed intra-aneurysmally, and the sheath was angled and delivered through the sheath into the atrial defect occluder. Intra-aortic angiography showed a complete sealing of the breach (**C**). After gradually increasing the pressure to 90/60 and 130/85 mmHg, the blocker was replaced and reimaged and placed in the normal position, and then the wire was rotated to release the blocker (**D,E**). Finally, the vehicle was observed for 5 min. A complete occlusion and good aortic valve function and occluder placement were confirmed (**F**).

Compared with open surgery, minimally invasive closure greatly lowers the risk of sudden death from a punctured pseudoaneurysm. Based on the results of the inflammatory indicator and blood cultures tests, which did not show significant bacterial infection, we utilized empirical antibiotics to manage the infection during hospitalization. After obtaining full informed consent, the patient chose minimally invasive repair of the pseudoaneurysm. After the patient was placed under general anesthesia and into the supine position, the right femoral artery was punctured with a 7F vascular sheath, and the left femoral artery was punctured with a 5F vascular sheath. The sinus duct junction was seen on the side of the greater curvature of the ascending aorta with a tumor-like deposit of contrast (Figures 2A,B). First, the blood pressure was lowered to approximately 70/40 mm Hg. The Lunderquist guidewire was then replaced and secured in the aneurysm. Then, the Cien-adjustable bend sheath was fed, the sheath was tilted, and the atrial defect occluder (42-28-32) was delivered through the sheath in the aneurysm. The anterior disc was opened and the fixed guidewire was gradually withdrawn posteriorly. The posterior disc was opened and the wire was delivered further forward without displacement of the occluder. Intra-aortic angiography showed a complete closure of the breach (Figure 2C). After gradually increasing the pressure to 90/60 and 130/85 mmHg, the blocker was placed in the normal position on separate angiograms, and the wire was then rotated to release the blocker (Figures 2D,E). Finally, the catheter sheath was retrieved in the descending aorta and observed for 5 min. A complete occlusion and good aortic valve function and occluder position were confirmed (Figure 2F). An intraoperative transesophageal ultrasound reassessment of the unaffected aortic valve function was performed (Supplementary Material S2).

**Figure 3:**
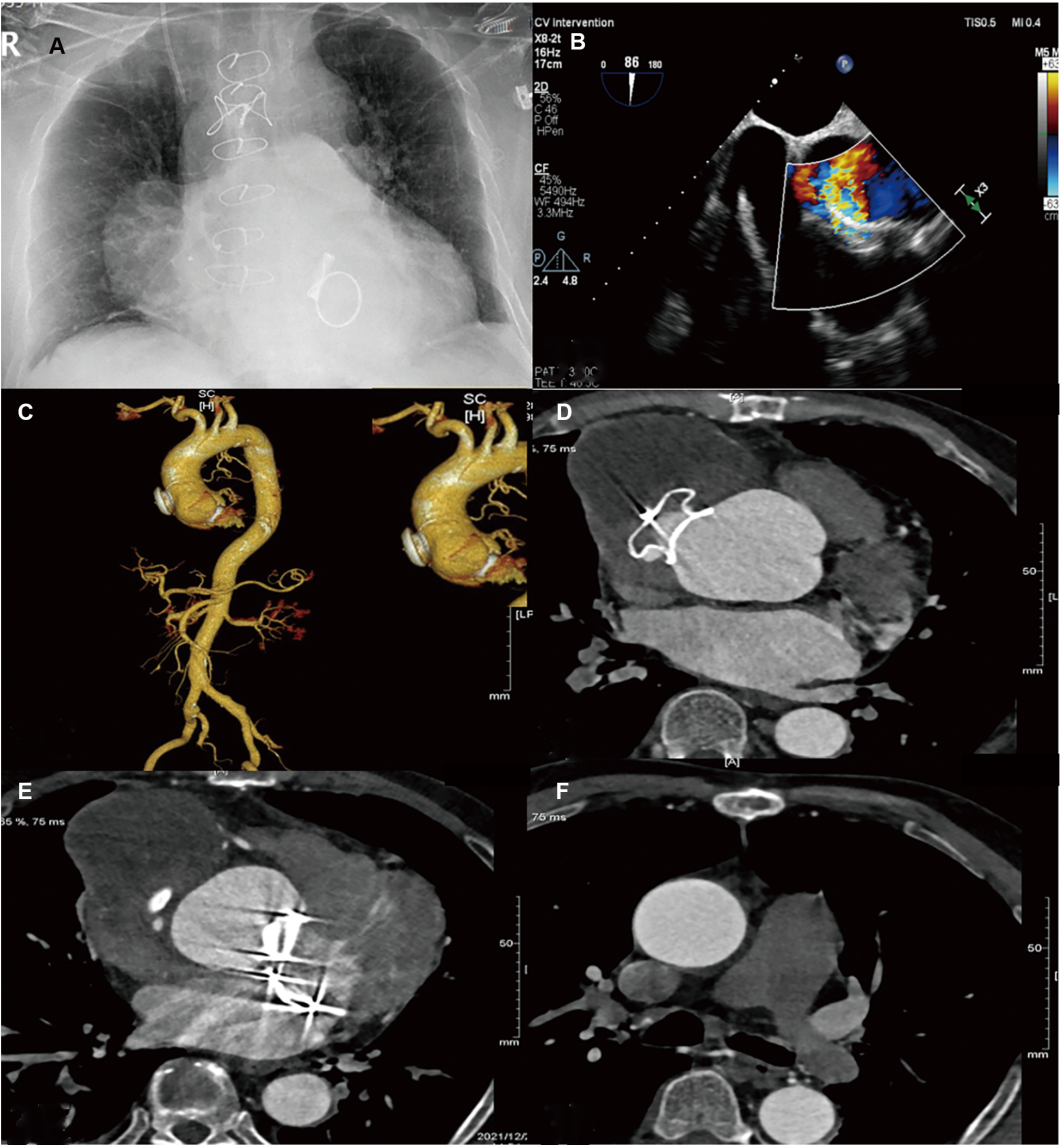
Preoperative: (**A**) Chest ray; (**B**) Cardiac ultrasound; (**C–F**) CT of the coronary artery + thoracic and abdominal aorta [(**C**) Front position; (**D**) Tumor waist; (**E**) Lower border of the tumor; (**F**) Pulmonary bifurcation)].

At the 3-month and 6-month follow-ups, our patient was found to have a good postoperative heart, liver, and kidney function. A postoperative chest x-ray showed no obvious abnormalities (Figure 3A). A postoperative echocardiography showed that the position of the AAP occluder was normal and the internal diameter of the ascending aorta had reduced (Figure 3B). A postoperative CT of the thoracic and abdominal aorta indicated that the occluder was in a normal position (Figures 3C–F).

## Discussion

During cardiac surgery, an ascending aorta pseudoaneurysm can lead to serious complications. Traditionally, surgery has been the recommended approach for the correction of thoracic pseudoaneurysms (4); however, it carries a risk of 7%–41% mortality and a very bad prognosis (5). As a result, percutaneous endovascular approaches have emerged as a viable alternative to traditional therapy (6).

Some studies have reported on the adoption of techniques such as a placement of a stent graft and transcatheter aortic valve implantation and the use of occluder devices vascular plugs to exclude pseudoaneurysm (7, 8). However, little information on whether endovascular therapy is suitable for progressive pseudoaneurysm patients is available (9). In our patient, a CT of the coronary arteries + thoracic and abdominal aorta provided us with accurate anatomical information. The aneurysm was located in the noncoronary sinus with a safe distance from the right and left coronary arteries, and it did not affect the coronary blood flow, given that the AAP neck was too large for the use of coils. In addition, due to the possibility of rupture of a huge pseudoaneurysm aneurysm at any time, the current instance was not appropriate for stent graft placement. Therefore, our case can be described as one of an asymptomatic patient with an expanding AAP. To the best of our knowledge, this is the largest device ever used for postsurgical pseudoaneurysm closure. A promising sign of progress toward final AAP closure was the reduced internal diameter of the ascending aorta shown on the 3-month and 6-month imaging follow-ups.

Minimally invasive surgery avoids a reoperation of the heart and reduces the risk of poor perfusion of all organs. In this case, the patient regained his postoperative cardiac function and had significantly improved liver and kidney function. Compared with open-heart surgery, minimally invasive surgery reduces the risk of surgery and complications such as intraoperative bleeding and infection. In this case, the patient was admitted with malnutrition and moderate anemia, and the pseudoaneurysm was in the active stage of rupture. We attempted to use antibiotics along with surgery to prevent infection. Our patient was discharged from the hospital one week after surgery and showed favorable outcomes in the follow-up; therefore, this treatment may provide a new option for the emergency treatment of patients with high-risk aortic pseudoaneurysms.

In conclusion, the procedure reduced not only the risk of death but also the incidence of complications. Our case may strengthen the understanding of surgeons about minimally invasive treatment of patients with high-risk pseudoaneurysms and inspire clinicians to choose a minimally invasive approach. Eventually, the shift in treatment philosophy may reduce perioperative mortality rates.

## Data Availability

The original contributions presented in the study are included in the article/**Supplementary Material**; further inquiries can be directed to the corresponding author.

## Supplementary material

**SUPPLEMENTARY IMAGE 1**

The progression of the patient's condition.

**SUPPLEMENTARY IMAGE 2**

The location of breaking mouth, the tumor neck was 4.01 cm.

**SUPPLEMENTARY IMAGE 3**

The breaking mouth from the right crown was 5.2 cm.

**SUPPLEMENTARY IMAGE 4**

The breaking mouth from the left crown was: 5.3 cm.

**SUPPLEMENTARY VIDEO 1**

An intraoperative transesophageal ultrasound reassessment of unaffected aortic valve function.

**SUPPLEMENTARY VIDEO 2**

Pre- and post-operative comparison of intraoperative transesophageal ultrasound of aortic valve function.

**SUPPLEMENTARY TABLE 1**

CARE Checklist.
