# Corrigendum: Minimally invasive closure of a progressive pseudoaneurysm of the ascending aorta: a case report

**DOI:** 10.3389/fcvm.2023.1232491

**Published:** 2023-07-12

**Authors:** Yuan Wu, Linglin Fan, Fei Liu, Hui Zhuang, Xijie Wu

**Affiliations:** ^1^ Department of Cardiac Surgery, Xiamen Cardiovascular Hospital of Xiamen University, School of Medicine, Xiamen University, Xiamen, China; ^2^ Department of Vascular Surgery, Xiamen Cardiovascular Hospital of Xiamen University, School of Medicine, Xiamen University, Xiamen, China

**Keywords:** aorta, pseudoaneursym, minimally invasive treatment, progressive, ascending

A Corrigendum on Minimally invasive closure of a progressive pseudoaneurysm of the ascending aorta: a case report by Wu Y, Fan L, Liu F, Zhuang H and Wu X. (2023) Front. Cardiovasc. Med. 10:1134196. doi: 10.3389/fcvm.2023.1134196

In the published article, there was an error in the Funding statement. An incorrect funding number was included.

The Funding statement has been updated:

This work was supported by grants from the Fujian Medical and Health Guiding Project (grant no. 2020D022).

The authors apologize for this error and state that this does not change the scientific conclusions of the article in any way. The original article has been updated.

