# Supplementary figures and images for: Minimally invasive closure of a progressive pseudoaneurysm of the ascending aorta: A case report
Source: Front Cardiovasc Med. 2023 Mar 20;10:1134196. doi: 10.3389/fcvm.2023.1134196 (PMC10064447; doi:10.3389/fcvm.2023.1134196)

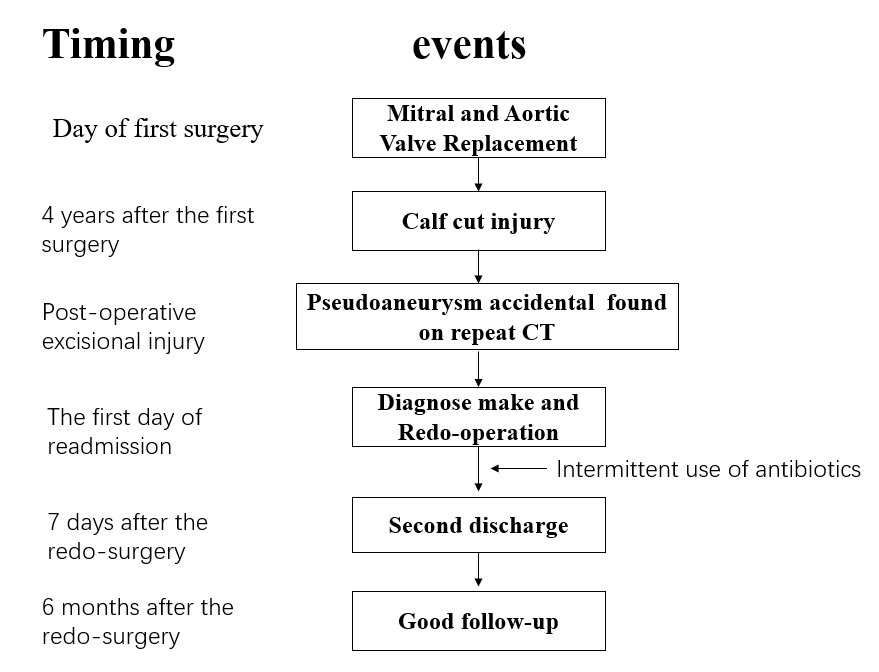

Supplement: Supplementary file 1 [file Image1.tiff]

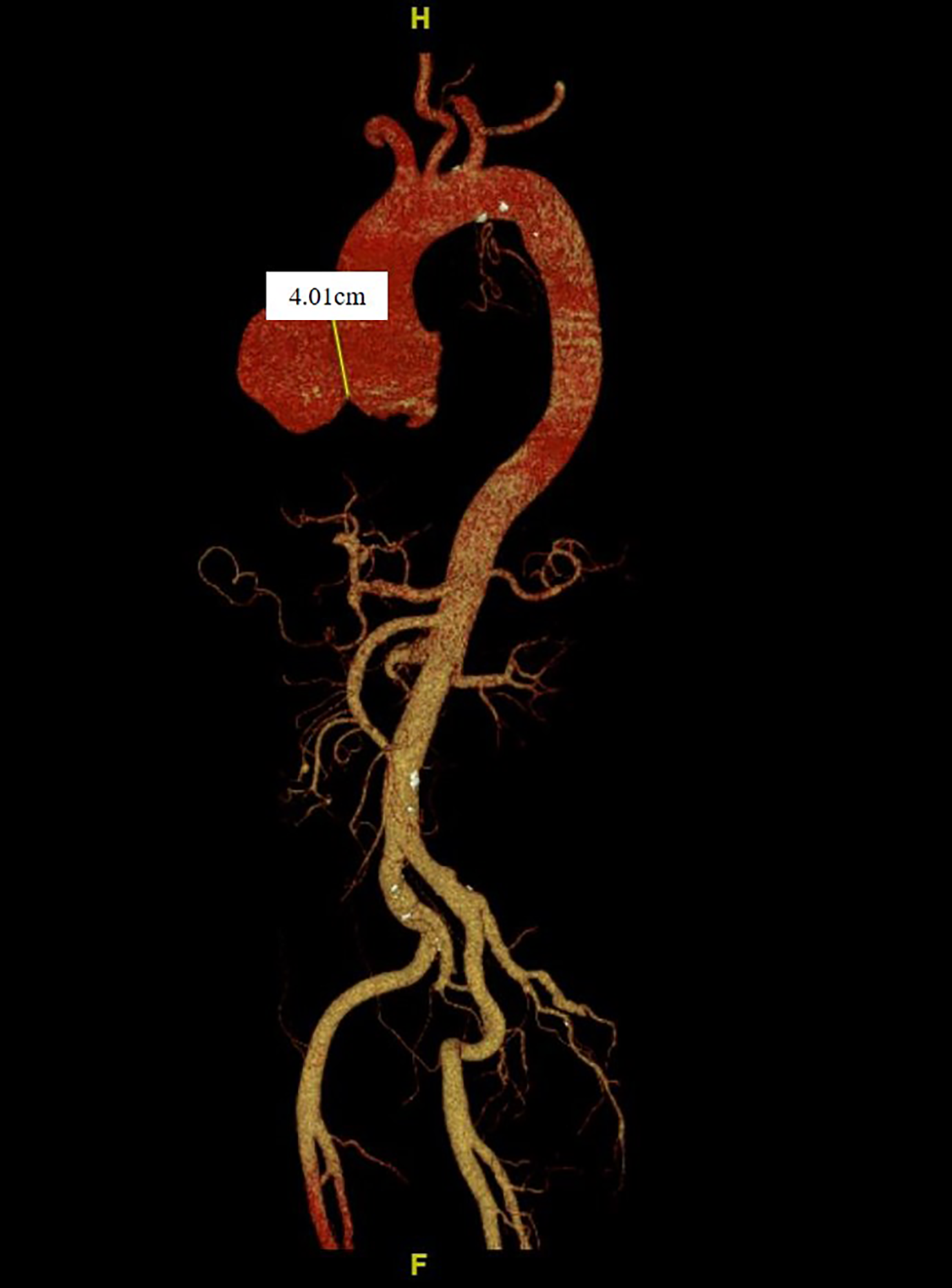

Supplement: Supplementary file 2 [file Image2.png]

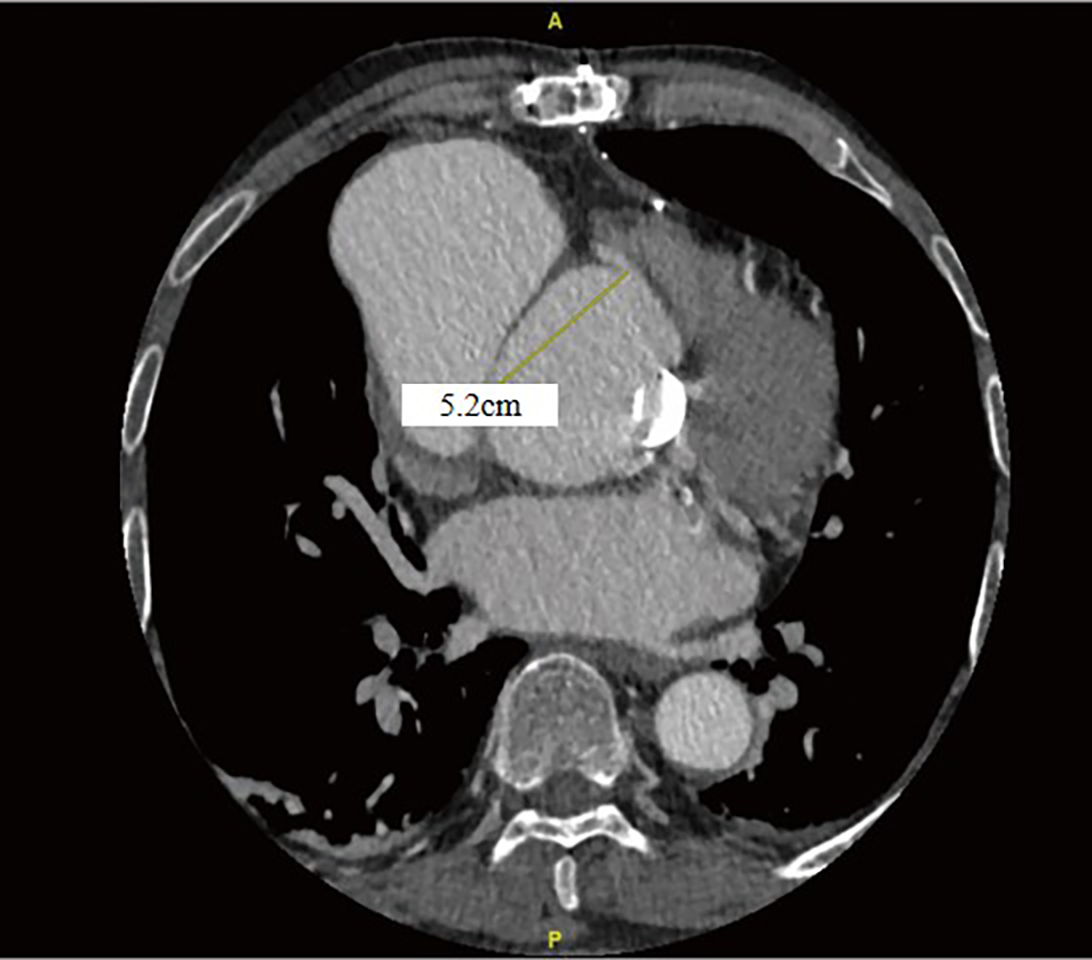

Supplement: Supplementary file 3 [file Image3.png]

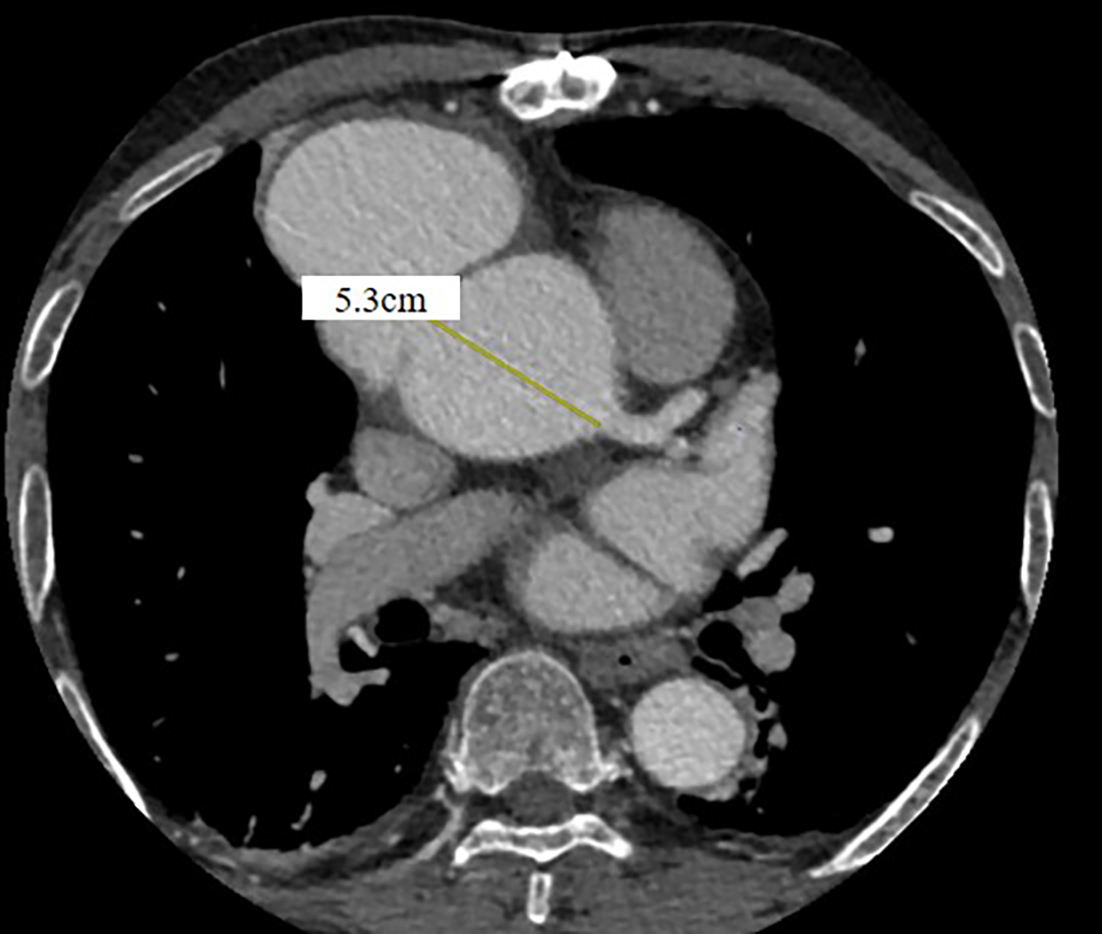

Supplement: Supplementary file 4 [file Image4.png]
